# High level transient production of recombinant antibodies and antibody fusion proteins in HEK293 cells

**DOI:** 10.1186/1472-6750-13-52

**Published:** 2013-06-26

**Authors:** Volker Jäger, Konrad Büssow, Andreas Wagner, Susanne Weber, Michael Hust, André Frenzel, Thomas Schirrmann

**Affiliations:** 1 Department of Molecular Structural Biology, Helmholtz-Zentrum für Infektionsforschung GmbH, Inhoffenstraße 7, Braunschweig 38124, Germany; 2 Department of Biotechnology, Technische Universität Braunschweig, Institute of Biochemistry, Biotechnology and Bioinformatics, Spielmannstr 7, Braunschweig 38106, Germany

**Keywords:** Recombinant Antibodies, Single Chain Fv, scFv-Fc, ImmunoRNase, Transient Mammalian Protein Production, Serum-free medium

## Abstract

**Background:**

The demand of monospecific high affinity binding reagents, particularly monoclonal antibodies, has been steadily increasing over the last years. Enhanced throughput of antibody generation has been addressed by optimizing in vitro selection using phage display which moved the major bottleneck to the production and purification of recombinant antibodies in an end-user friendly format. Single chain (sc)Fv antibody fragments require additional tags for detection and are not as suitable as immunoglobulins (Ig)G in many immunoassays. In contrast, the bivalent scFv-Fc antibody format shares many properties with IgG and has a very high application compatibility.

**Results:**

In this study transient expression of scFv-Fc antibodies in human embryonic kidney (HEK) 293 cells was optimized. Production levels of 10-20 mg/L scFv-Fc antibody were achieved in adherent HEK293T cells. Employment of HEK293-6E suspension cells expressing a truncated variant of the Epstein Barr virus (EBV) nuclear antigen (EBNA) 1 in combination with production under serum free conditions increased the volumetric yield up to 10-fold to more than 140 mg/L scFv-Fc antibody. After vector optimization and process optimization the yield of an scFv-Fc antibody and a cytotoxic antibody-RNase fusion protein further increased 3-4-fold to more than 450 mg/L. Finally, an entirely new mammalian expression vector was constructed for single step in frame cloning of scFv genes from antibody phage display libraries. Transient expression of more than 20 different scFv-Fc antibodies resulted in volumetric yields of up to 600 mg/L and 400 mg/L in average.

**Conclusion:**

Transient production of recombinant scFv-Fc antibodies in HEK293-6E in combination with optimized vectors and fed batch shake flasks cultivation is efficient and robust, and integrates well into a high-throughput recombinant antibody generation pipeline.

## Background

The increasing demand of monospecific detection reagents, particularly antibodies, for all human gene products is one of the biggest challenges to investigate the protein function and interaction [1-3]. Recently, we have demonstrated that phage display selection with our universal human naïve antibody gene libraries HAL4/7/8 can dramatically enhance the throughput of antibody generation and currently results in hundreds of new antibody clones per annum [4]. These recombinant antibodies have affinities in the low nanomolar range in some cases even in the subnanomolar range without any additional engineering [3]. Miniaturization and parallelization of panning and screening has been established in microtiter plate scale [5] and moves the bottleneck of the antibody pipeline towards the production and purification of milligram amounts of the individual antibodies in an application compatible recombinant format.

Single chain fragment variable (scFv) consisting only of the variable (V) regions of immunoglobulin (Ig) light (L) and heavy (H) chain connected by a soluble and flexible oligopeptide can be fused to the IgG Fc moiety. The resulting scFv-Fc format has properties of IgGs like bivalency, tag-free detection with standard secondary antibody conjugates and Fc-mediated effector functions [6,7]. We and others have previously shown that scFv-Fc antibodies can be used in all immunoassays including ELISA [8], immunoblot [9], antigen and antibody microarray [3], immunohistochemistry [10], immunofluorescence [7,11], quartz crystal microbalance immunosensors [12,13], surface plasmon resonance [8,14], and flow cytometry [8,15,16]. Moreover, scFv-Fc antibodies have been successfully used to neutralize viral, bacterial and fungal pathogens in vitro and in vivo [17-19] which suggests potential use in therapy.

Although production of scFv-Fc antibodies in yeast *Pichia pastoris* already achieved production levels of 10–30 mg/L [20], and recent developments with glycoengineered yeasts [21] for commercial antibody production [22] and high throughput screening [23] are promising, mammalian cell antibody expression systems are still being advanced regarding production yields and product quality [24]. Today, almost all therapeutic antibodies are produced in mammalian cells because their advanced folding, secretion and post-translational apparatus is best suited to produce antibodies indistinguishable from those produced in the human body with least concerns for immunogenic modifications. Industrial IgG production levels in Chinese hamster ovary (CHO) cells reached about 5 g/L some years ago [25] whereas today titers often exceed 12 g/L as result of a steadily ongoing progress of mammalian cell culture technology which is mainly due to improved high producer cell lines, optimized serum-free production media as well as optimized and prolonged production processes at very high cell densities. The highest IgG titer has been reported in the human embryonic retinal cell line Per.C6 (Crucell, Leiden, The Netherlands) with 27 g/L in a perfusion bioreactor. Although the generation of high producer cell lines has been dramatically improved and accelerated [26,27], it is still too expensive, time-consuming and laborious for research applications, particularly if large numbers of individual antibodies have to be produced.

Here, transient and semi-stable mammalian antibody expression is much more suitable because it allows fast and parallelized production without any need to generate producer cell lines [28]. Moreover, transient mammalian antibody production can be scaled up by employing batch or fed-batch bioreactor processes to more than 150 liter production volumes [29]. Therefore, transient antibody production is suitable for small scale production for antibody screening [30], but also capable to generate even grams of antibodies [31-33].

Particularly, human embryonic kidney (HEK) 293 cell lines have been used for transient protein expression because they can be very efficiently transfected with plasmid DNA. This cell line was generated from embryonal tissue by transformation with sheared adenovirus 5 DNA. Some derivatives were further transformed either with the simian virus 40 (SV40) large T antigen, termed HEK293T, or with the Epstein Barr virus (EBV) nuclear antigen 1 (EBNA1), termed HEK293E, in order to mediate semi-stable episomal propagation of vectors containing an origin of replication (ori) of SV40 or EBV, respectively. Transient transfection of plasmid DNA in HEK293 cells can be performed by calcium phosphate transfection [34], cationic liposomes and polymers. The cationic polymer polyethyleneimine (PEI) combines highly efficient plasmid delivery with low cytotoxicity and simple handling [35-37]. PEI can be used in serum-containing as well as serum-free media [38] and is compatible with upscaling of the production volume [39-42]. The large number of protonable amino groups of PEI results in its cationic charge, which is responsible for DNA complexation [35,36]. These protonable amino groups also provide high pH buffering capacity which seems to protect the DNA from degradation in endosomal compartments and to mediate an early escape of DNA/PEI complexes from lysosomes probably by the so-called proton sponge effect [43]. According to the proton sponge hypothesis, the buffering capacity of PEI leads to osmotic swelling and rupture of endosomes, resulting in the release of the complexed DNA into the cytoplasm.

In this study, we optimized mammalian expression vectors for single step in frame cloning of scFv fragments from our universal antibody phage display libraries HAL4/7/8 [4] into the IgG-like scFv-Fc format. In combination with transient production using the HEK293-6E cell line, a variant genetically modified with a truncated version of EBNA1 which grows in suspension and chemically defined serum-free medium [44,45], we achieved volumetric yields of more than 0.5 g/L for recombinant antibodies as well as for an immunoRNase fusion protein by simple shake flask cultivation. We demonstrate that transient scFv-Fc production in HEK293-6E is robust, versatile and highly efficient and can overcome the bottleneck of antibody production in a high throughput in vitro antibody selection pipeline.

## Results

### Construction and test of vectors for transient scFv-Fc and immunoRNase expression in HEK293 cells

**Figure 1:**
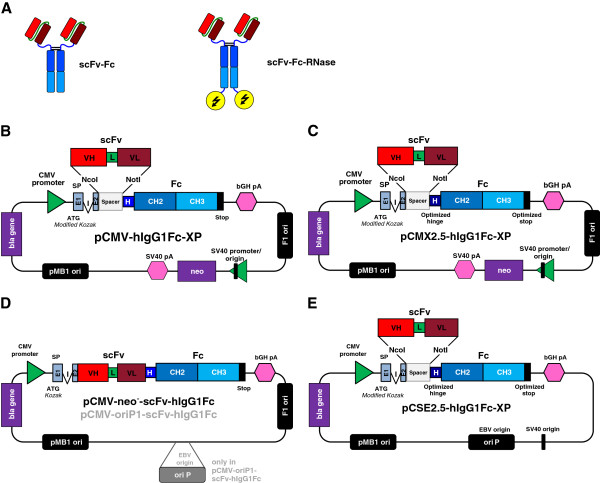
**Development of vectors for transient antibody expression in HEK293 cell lines. A**) Illustration of the structure of scFv-Fc and scFv-Fc-RNase constructs. **B**) The vector pCMV-hIgG1Fc-XP based on pCMV/myc/ER backbone which contains a neomycin phosphotransferase (neo) selection marker under control of a simian virus (SV) 40 promoter (with SV40 ori) and poly A (pA) signal. The antibody gene expression cassette consists of a modified untranslated 5’ region and ribosomal binding site (modified KOZAK sequence), mouse heavy Ig chain leader comprising two exons (E1 and 2, light blue) interrupted by one intron (I) for stabilized expression, followed by a spacer sequence (grey) flanked unique NcoI/NotI sites for single step in frame cloning and fusion with following constant hinge, CH2 and CH3 regions of human IgG1 Fc gene fragment (dark blue). The gene expression cassette is driven by a CMV promoter and terminated by bovine growth hormone (bGH) poly adenylation signal (pA). **C**) pCMX2.5-hIgG1Fc-XP has a modified hinge without genetic upper hinge of the IgG1 heavy chain and optimized translation termination sequence at the 3’ of the Fc gene fragment. **D**) pCMV-neo^-^ and pCMV-oriP1 test vectors containing CD30 specific scFv-Fc or scFv-Fc-RNase were generated from the parental pCMV vectors [11] by deleting the complete neo expression cassette or replacing it by a short EBV oriP variant. **E**) The expression vector pCSE2.5-hIgG1Fc-XP was newly generated and comprises all advantages for transient expression of the previous vectors including the optimized hinge and stop sequences of pCMX2.5, no selection marker expression cassette, minimal SV40 ori and short EBV oriP for episomal replication in SV40-large T antigen or EBNA1 positive mammalian expression cell lines.

**Figure 2:**
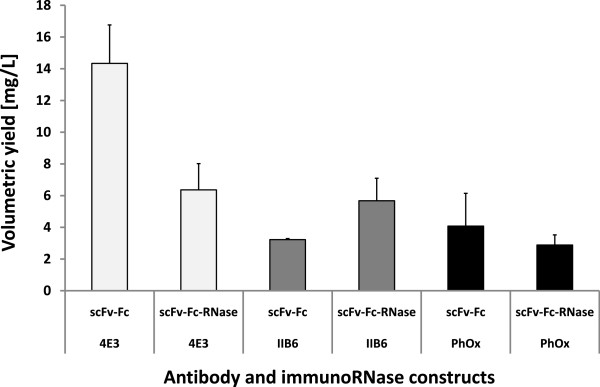
**Transient expression of different scFv-Fc and scFv-Fc-RNases in HEK293T.** Different scFv-Fc antibodies and the corresponding immunoRNase fusion proteins were cloned into the pCMV vector and transiently expressed in HEK293T cells. Volumetric yields were measured with a human IgG/Fc capture ELISA. Mean values and standard deviations were determined by at least 3 independent experiments.

Transient expression in HEK293 cell lines was initially tested with two previously described pCMV vectors encoding either the CD30 specific scFv-Fc-4E3 antibody or the corresponding immunoRNase (Figure 1A), respectively [11]. We reproducibly achieved volumetric yields of 14 mg/L for the scFv-Fc and 7 mg/L immunoRNase (Figure 2). Transient transfection was performed with PEI which allowed very high transfection efficiencies of more than 70% in HEK293T cells, as previously described [38]. Transfection with PEI resulted in higher antibody yields than several commercial polymers- or liposome-based transfection reagents, which was attributed to PEI’s lower cytotoxicity (data not shown). The optimal transfection conditions were previously determined with 1 μg DNA to 8 μg PEI per mL cell culture [38]. Additional scFv-Fc antibodies and immunoRNase proteins were also tested in the same vector system and reached 4 to 6 mg/L in HEK293T cells (Figure 2).

In order to facilitate cloning of single chain antibody fragments from phage display antibody gene libraries such as HAL4/7/8 [4] into the IgG-like scFv-Fc format, the new vector pCMV-hIgG1Fc-XP (Figure 1B) was generated from the pCMV/myc/ER-derived vector pCMV-HC by modifying the 5′ untranslated region, the ribosomal binding site [46] and by introducing a Nco/NotI compatible multiple cloning site. The original NcoI site at the start codon was removed by transversion from cytidine to adenine at position −2 in order to preserve high translation initiation efficiency because it is described as second abundant nucleotide at this position in vertebrate mRNAs [47]. The residual secretory mouse Ig heavy chain signal peptide consisting of two exons and one intron for stabilized expression was retained. Following, a new spacer sequence was introduced which is flanked by the new unique NcoI/NotI cloning sites allowing single step in frame cloning of scFv antibody fragments from the HAL4, 7 and 8 phage display libraries in fusion with the gene fragments encoding aminoterminal mouse Ig heavy chain signal peptide and carboxyterminal hIgG1-Fc moiety. All vectors containing this NcoI/NotI cloning cassette are designated with “XP”. The pCMV-hIgG1Fc-XP vectors were further optimized at the 3′ stop translation sequences of the Fc gene fragment (Table 1) and tested by transient expression in adherent HEK293T cells. The sequence of the original BamHI/XbaI cloning site and the upstream opal stop codon was prone to mutations in the stop codon to TTA. Therefore, we eliminated the BamHI site in direct vicinity of the stop codon and tested the *opal* stop codon (TGA) with one, five or 14 downstream nucleotides from the human genomic Ig sequence (pCMV2.1-hIgG1Fc-XP, pCMV2.2-hIgG1Fc-XP, pCMV2.3-hIgG1Fc-XP) as well as with one adenine (pCMV2.4-hIgG1Fc-XP) followed by the XbaI site (Table 1). The adenine as fourth nucleotide is the most frequent in translation termination signals of eukaryotic genes and provides very low read through of the ribosome [48,49]. We also tested the ochre stop codon (TAA) followed by one adenine (pCMV2.5-hIgG1Fc-XP). Moreover, we constructed two vectors with an Fc gene fragment whose 3′ terminal lysine codon was either deleted (pCMV3.2-hIgG1Fc-XP) or replaced by a BamHI site reconstituting codons for the two amino acids glycine and serine (pCMV4.2-hIgG1Fc-XP), respectively (Table 1). The translation termination sequence of the latter two vectors matched those from vector pCMV2.2-hIgG1Fc-XP.

**Table 1:** pCMV-hIgG1Fc-XP vectors with modified 3′ translation termination sequences

**pCMV**	**5→3’**	**Features**
-	TCTCCCTGTCCCCGGGTAAATGA**G**GATCCTCTAGAAGCT	*opal*^a^, BamHI, XbaI
2.1	TCTCCCTGTCCCCGGGTAAATGA**G**TCTAGAAGCT	*opal* + 1 nt, XbaI
2.2	TCTCCCTGTCCCCGGGTAAATGA**G**TGCGTCTAGAAGCT	*opal* + 5 nts, XbaI
2.3	TCTCCCTGTCCCCGGGTAAATGA**G**TGCGACGGCCGGCTCTAGAAGCT	*opal* + 14 nts, XbaI
2.4	TCTCCCTGTCCCCGGGTAAATGA**A**TCTAGAAGCT	*opal* + dA, XbaI
2.5	TCTCCCTGTCCCCGGGTAAATAA**A**TCTAGAAGCT	*ochre* + dA, XbaI
3.2	TCTCCCTGTCCCCGGGATGA**G**TGCGTCTAGAAGCT	ΔK330^b^, *opal* + 5 nts, XbaI
4.2	TCTCCCTGTCCCCGGGATCCTGA**G**TGCGTCTAGAAGCT	K330S^b, c^, BamHI, *opal* + 5 nts, XbaI

Abreviations: *nt*, desoxynucleotide(s) derived from human IgG gene sequences; dA, desoxy-adenine.

underlined … stop codon, bold letter… desoxynucleotide downstream from the stop codon also termed as “forth nucleotide of the stop codon” is important efficient for translation termination.

^a^ Frequent mutations of *opal* (TGA) to TTA were observed which might be due to the immediately following BamHI and XbaI sites.

^b^ Numbering according to Uniprot: P01857.

^c^ Additional BamHI fusion site.

**Figure 3:**
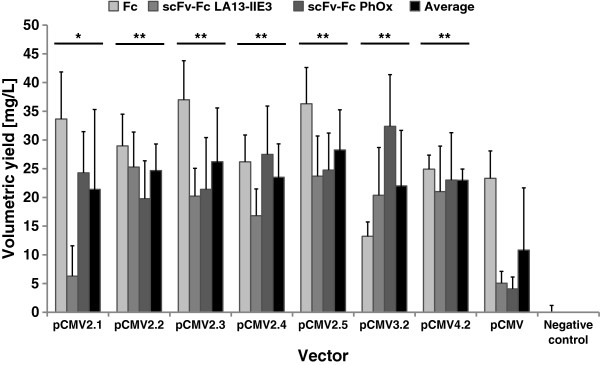
**Test of different translation termination sequences for transient expression in HEK293T cells.** A number of pCMV vectors were generated containing the *opal* stop codon and the following one (pCMV2.1), five (pCMV2.2), or 14 nucleotides (pCMV2.5) derived from genomic human Ig sequence or adenine as “fourth” stop codon nucleotide (pCMV2.4). Moreover, we also tested vector constructs containing an *ochre* stop codon followed by an adenine (pCMV2.5) and pCMV2.2 variants where the 3' terminal lysine codon was deleted (pCMV3.2) or replaced by a *BamH*I cloning site encoding the amino acids GS instead (pCMV4.2). Transient productions were done in HEK293T cells and tested by human IgG/Fc capture ELISA 48 h after transfection. Mean production levels and standard deviations of three productions are given. Cells transfected with the YFP reporter gene are used as negative control for the ELISA. Statical analysis was performed by comparing each vector with the vector pCMV using a t-test. Significance is indicated (* … p < 0.05 and ** p < 0.01). ND not determined.

Transient expression of pCMV2.1-, pCMV2.2-, pCMV2.3-, pCMV2.4-, pCMV2.5-, pCMV3.2- and pCMV4.2-hIgG1Fc-XP vectors in HEK293T cells resulted in a 1.5- to 3-fold higher average scFv-Fc antibody production level than the original pCMV-hIgG1Fc-XP vector depending from the expressed protein (Figure 3). The vector pCMV2.1-2.5 and pCMV4.2 achieved with little exceptions very similar expression levels of 15–35 mg/L Fc and scFv-Fc proteins when measured 48 h after transfection. We chose pCMV2.5-hIgG1Fc-XP showing the best average results for further vector development and eliminated the genetic upper hinge region of hIgG1 to avoid unspecific binding of the unpaired cysteine which normally forms the interchain disulfide bond between light and heavy chain in IgG1 and which is not required in scFv-Fc antibodies. The resulting new pCMX2.5-hIgG1Fc-XP vector (Figure 1C) achieved comparable or slightly higher production levels for scFv-Fc antibodies in HEK293T cells than the corresponding pCMV2.5-hIgG1Fc-XP vector (data not shown).

### Transient expression of standard mammalian expression vectors in HEK293-6E cells

**Figure 4:**
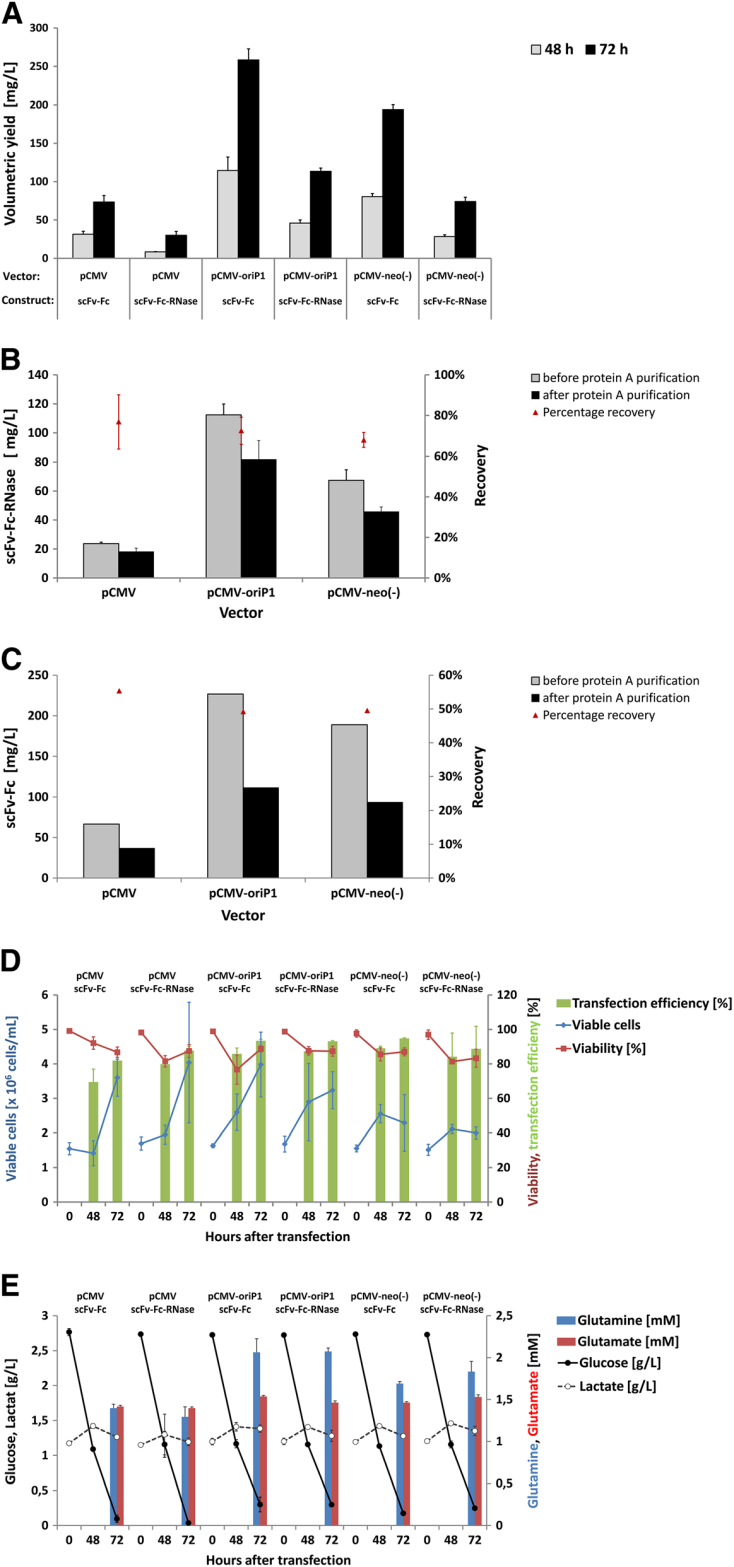
**Test of optimized antibody expression vectors in HEK293-6E cells.** Three different vectors pCMV-, pCMV-oriP1- and pCMV-neo^-^ -scFv-Fc-4E3 encoding a CD30 specific scFv-Fc antibody, and another three vectors pCMV-, pCMV-oriP1- and pCMV-neo^-^ -scFv-Fc-RNase-4E3 encoding a CD30 specific immunoRNase, respectively, were transiently transfected into HEK293-6E cells in 50 mL scale in shake flasks. Production of each vector and each construct were performed as triplicates. **A**) After 48 and 72 h, volumetric yields of scFv-Fc and scFv-Fc-RNase were tested from production supernatants by human IgG/Fc capture ELISA. **B-C**) After 72 h of scFv-Fc (D, triplicate samples individually purified) and scFv-Fc-RNase (E, triplicate samples combined prior purification) were purified by protein A affinity chromatography and volumetric yields were calculated. Percentage recovery is calculated in comparison to the volumetric yields measured from supernatants (**A**). **D**) Several production parameters were tested like transfection efficiency (co-transfection with an GFP expressing vector), viable cell number and viability as well as **E**) concentrations of glucose, L-lactate, L-glutamine and L-glutamate.

First transient expression studies in the HEK293-6E cell line [41] were conducted by using mammalian expression vectors pCMV-scFv-hIgG1Fc-4E3 and pCMV-scFv-hIgG1Fc-RNase-4E3 encoding a CD30 specific scFv-Fc antibody and the corresponding immunoRNase fusion protein [11]. Optimal DNA to PEI ratio was previously determined with 1:2 to 1:8 and an optimal plasmid DNA concentration of 0.5 to 1 μg/mL [38]. We used standard transfection conditions of 1 μg plasmid DNA and 2.5 μg PEI per mL for all further experiments and achieved volumetric yields of 30 to 40 mg/L scFv-Fc-RNase and 70 to 80 mg/L scFv-Fc, respectively, already after 3 days of production (Figure 4A), and 60–70 mg/L scFv-Fc-RNase and 120 to 140 mg/L scFv-Fc, respectively, after 5 days of production [38]. Although the volumetric yields in HEK293-6E cells were about 10-fold higher than in adherent HEK293T, the 5 days product accumulation and the higher cell densities have to be taken into account. Transfection efficiencies measured with a fluorescent reporter protein usually varied between 40-50% [38], but we also observed transfection efficiencies of up to 90% which seem to depend on the number of cell passages and the general condition of the cells.

In order to verify that the transfection of cells was completed by PEI:DNA complexes after 48 hours, an additional experiment was performed where culture supernatant was collected 48 hours post transfection and used in combination with fresh non-transfected cells. No additionally transfected cells were observed in these cultures as measured by flow cytometry (data not shown).

### Construction and optimization of vectors for transient expression in HEK293-6E cells

Since the pCMV or pCMX vector backbones are not compatible to the episomal replication machinery in the HEK293-6E cells which express a truncated version of EBNA1, vector optimization was done by generating two variants of both vectors, pCMV-scFv-hIgG1Fc-4E3 (6391 bp) and pCMV-scFv-hIgG1Fc-RNase-4E3 (6769 bp), encoding a CD30 specific scFv-Fc antibody and the corresponding scFv-Fc-RNase variant. First, the complete neomycin phosphotransferase (neo) selection marker expression cassette including its SV40 promoter/ori and poly-A signal as well as the F1 origin from the vector backbone were eliminated resulting in plasmids pCMV-neo^-^-scFv-Fc and -scFv-Fc-RNase comprising only 4338 and 4715 bp (Figure 1D), respectively. Then, a short version of the EBV oriP was introduced into these vectors resulting in the vectors pCMV-oriP1-scFv-Fc and pCMV-oriP1-scFv-Fc-RNase with a size of 5327 and 5705 bp (Figure 1D), respectively.

The HEK293-6E expression of pCMV, pCMV-neo^-^ and pCMV-oriP1 containing scFv-Fc- and scFv-Fc-RNase-4E3, respectively, was done in three independent samples in 25–50 mL scale in shake flasks. After 3 day of production the small pCMV-neo^-^ vector missing the selection marker already resulted in a 2.5-fold increase of volumetric yields for scFv-Fc and scFv-Fc-RNase, respectively, compared to the parental pCMV vectors (Figure 4A). The pCMV-oriP1 vectors containing an EBV oriP with shortened spacer sequence between FR and DS element even achieved 3.5 fold higher yields for both constructs compared to the pCMV vectors (Figure 4A). Production supernatants were harvested and purified by protein A affinity chromatography. The scFv-Fc-RNase protein samples were separately purified and achieved recovery rates of about 80% (Figure 4B). In contrast, supernatants of triplicate productions of each scFv-Fc antibody expression vector were combined prior purification which resulted in a lower recovery rate of about 50% probably due to the lower efficacy of the 1 mL Bio-Scale Mini UNOsphere SUPrA Cartridges (binding capacity of human IgG1 ca. 20 mg) when applying more than 10 mg scFv-Fc antibody (Figure 4C). Nevertheless, transient production of scFv-Fc and scFv-Fc-RNase using the pCMV-oriP1 vector resulted in the highest amount purified, followed by the pCMV-neo^-^ vector and the parental pCMV vectors. After transfection, the concentration of viable cells increased from 1.5×10^6^ cells per mL to up to 4×10^6^ cells per mL in the following 3 days for the pCMV and pCMV-oriP1 vectors, whereas pCMV-neo^-^ transfected cells did not exceed 2.5×10^6^ cells per mL (Figure 4D). After 3 days of production, viability in samples was 85 to 90% (Figure 4D). Transfection efficiencies tested by co-transfection with a fluorescent reporter plasmid were 75 to 90% (Figure 4E). In addition, several metabolic parameters were tested in this experiment. The initial glucose concentration of about 3 g/L was almost entirely consumed after 3 days whereas L-lactate concentrations remained at an almost constant level throughout the 3 days production period (Figure 4E). L-glutamine (initially at 7.5 mM) and L-glutamate concentrations were tested after 72 h and found at about 1.5 and 2 mM, respectively (Figure 4E). This early depletion of important nutrients of the cellular energy metabolism combined with the fact that episomal plasmid replication will enable cells for a sustained recombinant protein production suggests that the original protocol provided from BRI has a potential for further optimization.

### Optimization of scFv-Fc and immunoRNase production in HEK293-6E cells

**Figure 5:**
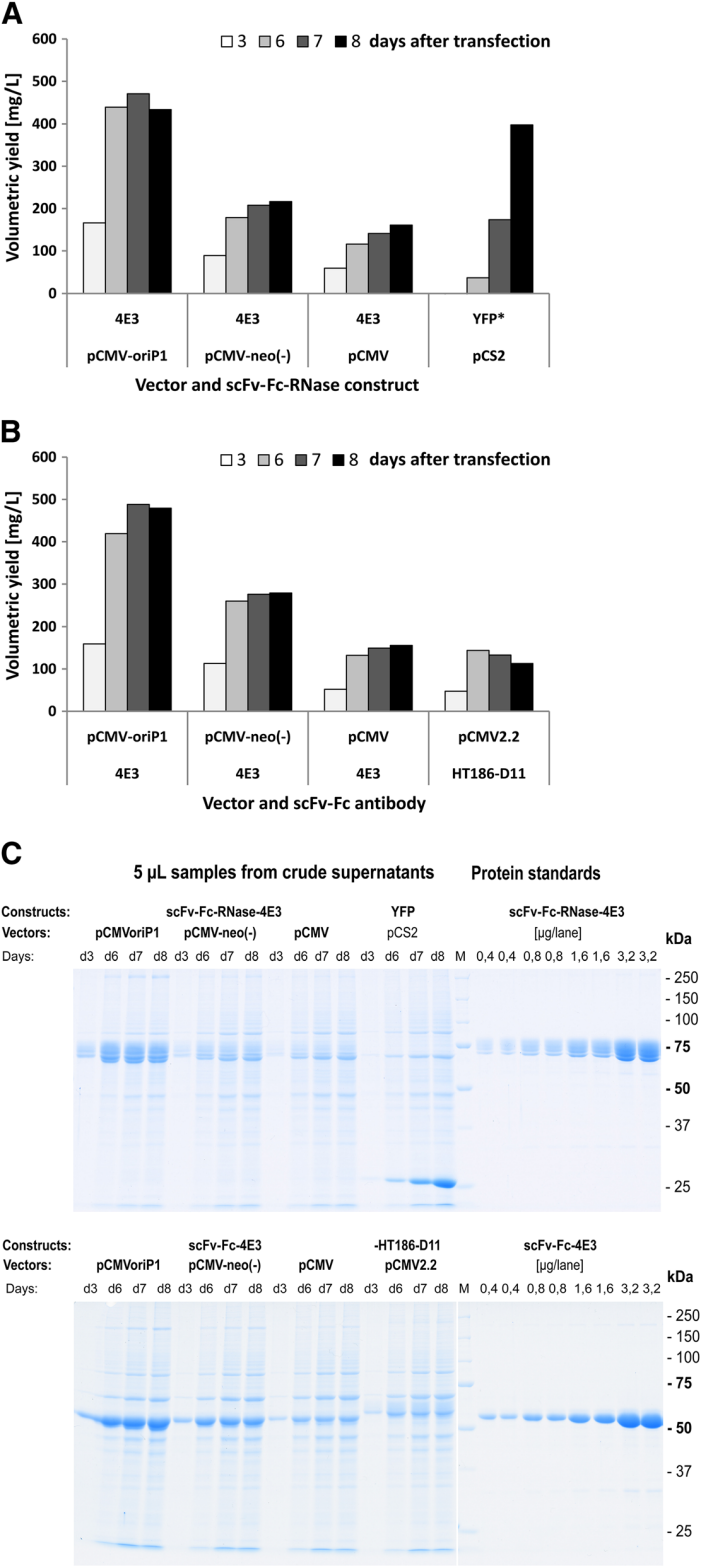
**Prolonged transient protein production in HEK293-6E cells. A**) and **B**) The vectors pCMV-oriP1, pCMV-neo^-^ and pCMV containing CD30 specific scFv-Fc- and scFv-Fc-RNase-4E3 constructs, respectively, were transiently transfected into HEK293-6E cells in 25 mL scale in shake flasks. In addition, pCMV2.2-scFv-Fc with the MUC1-specific affinity maturated scFv HT186-D11 [8] and a plasmid pCS2-venus expressing the yellow fluorescent reporter protein (YFP, venus) were transfected in parallel attempts. Production samples were taken 3, 6, 7, and 8 days after transfection. **A**) Volumetric yields of scFv-Fc and scFv-Fc-RNase were tested by gel densitometry. **C**) SDS-PAGE was performed with 5 μL of each supernatant under reducing conditions. Gels were stained with coomassie (G250). Purified scFv-Fc- and scFv-Fc-RNase-4E3 were used as standards for gel densitometry. YFP was compared to the scFv-Fc standard.

In the original protocol and in the initial experiments we have seen that best yields were obtained after at least 5 days production. Therefore, the production after transfection with pCMV, pCMV-neo^-^ and pCMV-oriP1 with scFv-Fc- and scFv-Fc-RNase-4E3 was prolonged (Figure 5). For this purpose, cultures were fed 48 h post transfection with 0.5% trypton TN1 plus one additional volume of fresh F17 medium. Compared to the 3 days protocol maximum yields increased more than 100% after 6 days for most vector constructs whereas a further extension of production beyond did not result in any significant additional improvement. Highest production levels were achieved after 6–7 days for the vector pCMV-oriP1 with more than 450 mg/L for both scFv-Fc-4E3 (Figure 5A) and scFv-Fc-RNase-4E3 (Figure 5B). For comparison, scFv-Fc-4E3 and the MUC1-specific scFv-Fc-HT186-D11 [8] expressed by the original vector pCMV yield in about 120 mg/L (Figure 5B).

Interestingly, YFP expressed by a pCMV-like vector in a parallel sample, was only slightly released into the supernatant after 6 days which can be seen as slight 27 kDa protein band in SDS-PAGE (Figure 5C). However, YFP release into the supernatant increased to 150 to more than 350 mg/L after 7 to 8 days, respectively, which is associated with the increasing cell lysis and release of cytoplasmic proteins.

**Figure 6:**
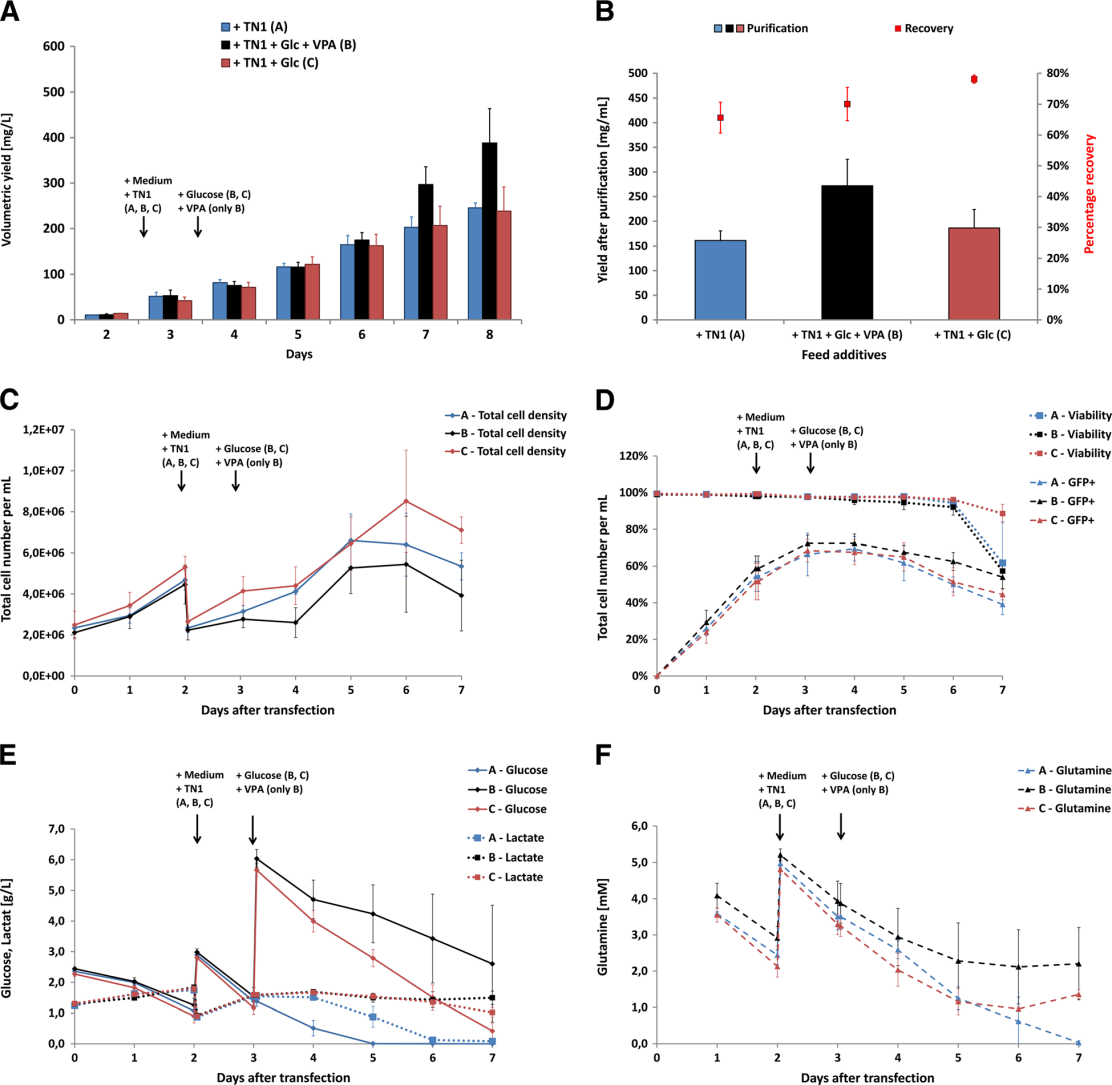
**Fed-batch optimization of transient scFv-Fc-RNase protein production in HEK293-6E cells.** The vector pCMV-oriP1-scFv-Fc-RNase-4E3 was transiently transfected into HEK293-6E cells in 25 mL scale in shake flasks. One volume of fresh medium and 0.5% (w/v) tryptone TN1 was fed 48 h after transfection to all groups (group **A**, **B**, **C**). After 3 days two groups (group **B**, **C**) were fed with glucose and one group with additional 3.75 mM valproic acid (VPA, only group **C**). **A**) Volumetric yields of scFv-Fc-RNase protein were measured from supernatant samples taken from day 2 to 8 after transfection. **B**) ScFv-Fc-RNase was purified by protein A affinity chromatography from production supernatants harvested 8 days after transfection. The yield was calculated from total amount of purified protein and the final production volume. Protein recovery was determined as ratio of scFv-Fc-RNase after and before purification. **C**) Cell number, **D**) viability and and transfection efficiency (GFP co-transfection), **E**) Glucose and lactate as well as **F**) L-glutamine and glutamate were measured during the production until day 7. All groups were performed in independent triplicate samples. Mean values as well as standard deviations are given.

In addition to the standard fed of one volume medium and trypton TN1 48 h after transfection, additional supplementation with glucose alone and in combination with the histone deacetylase inhibitor VPA were tested (Figure 6). The fed of glucose together with VPA significantly increased the scFv-Fc-RNase production in two of three independent samples, whereas glucose alone did not result in any higher yield in comparison to the standard medium TN1 supplementation (Figure 6A, B). Interestingly, VPA treatment led to lower cell growth with maximum cell concentration of 4×10^6^ cells/mL 6 days after transfection, whereas the other two groups reached 6-8×10^6^ cells/mL (Figure 6C). From day 6 to 7 of production, the cell concentration began slightly to decrease in all groups associated with a reduced viability <70% except in the VPA treated group where the viability still remained >90% (Figure 6D). After 3 to 4 days of production, the ratio of GFP co-transfected cell decreased in all groups indicating that cells stressed by transgene expression were more prone to cell death or lysis than non-transfected cells (Figure 6D). A more detailed look into the metabolic parameters (Figure 6E, F) revealed that the standard medium plus TN1 fed prevented complete glucose consumption only for 5 days and lactate was also consumed one day later (Figure 6E). Nevertheless, the product yield still doubled from day 5 to 8. Additional glucose supplementation delayed complete glucose metabolization within the tested 7 days production period without increasing the protein production (Figure 6F). In contrast to glucose, L-glutamine was not completely metabolized during 7 days of production in all groups. Additional VPA treatment led to clearly lower glucose and L-glutamine consumption of all groups.

### Generation of an optimized library compatible scFv-Fc vector for HEK293-6E expression

**Figure 7:**
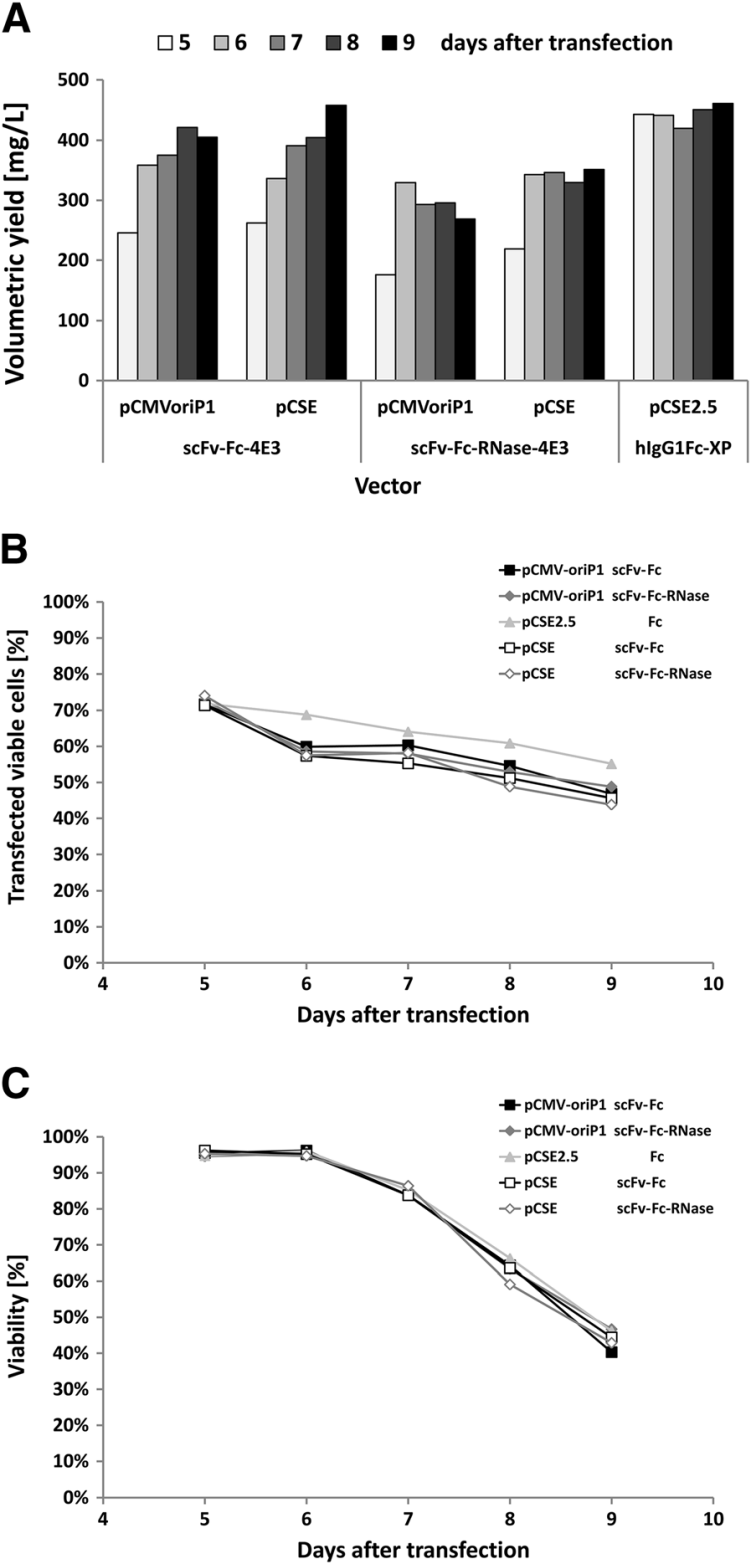
**Comparison of the optimized expression vectors for transient production in HEK293-6E cells.** Two pCMV-oriP1 and pCSE vectors expressing the CD30 specific antibody scFv-Fc-4E3 and the corresponding immunoRNase, respectively, as well as the pCSE2.5-hIgG1-XP vector that expresses only the hIgG1-Fc fragment were transiently transfected into HEK293-6E cells in 25 mL scale and shake flasks. After 48 h one volume fresh medium and 0.5% tryptone TN1 was added. After 5 days additional 0.5% TN1 was fed. **A**) Volumetric yields were determined by gel densitometry from supernatants. **B**) Transfection efficiency was measured by co-transfection with YFP reporter plasmid (1/20 of the total plasmid DNA) using flow cytometry. **C**) Viability of the cells was also measured by flow cytometry with PI staining.

In order to obtain an optimal transient mammalian expression vector that comprises all advantages of the previous vectors, a new vector was constructed. First, a minimal backbone of the vector pCMX2.5-hIgG1Fc-XP was generated. Then, the entire antibody gene expression cassette of pCMX2.5-hIgG1Fc-XP comprising CMV promoter and enhancer, the 5′untranslated region including the modified ribosomal binding site, the gene sequences for the murine immunoglobuline signal peptide including the intron, the spacer containing library compatible multiple cloning sites and the hIgG1 Fc moiety were introduced. In a third step, the bGH poly A signal with reduced adjacent spacer sequences, a minimal SV40 *ori*[50] and a shortened variant of the EBV *ori*P were introduced. The gene expression cassettes of the CD30 specific scFv-Fc-4E3 antibody and the scFv-Fc-RNase-4E3 immunoRNase were introduced in this vector in order to obtain the plasmids pCSE-scFv-hIgG1Fc-4E3 and pCSE-scFv-hIgG1Fc-RNase-4E3, respectively. Transient production in HEK293-6E was tested by standard supplementation with one volume medium and 0.5% TN1 hydrolysate and achieved volumetric yields of 300 to 400 mg/L of scFv-Fc-RNase and scFv-Fc, respectively, which were similar or even slightly higher than those obtained by the corresponding pCMV-oriP1 test vectors transfected in parallel samples (Figure 7A). Highest production levels were obtained after 6 days when the viability of the cells was still >90% (Figure 7C). Transfection rates were identical for all these vectors (Figure 7B).

In order to obtain a universal library compatible vector for the expression of scFv-Fc antibodies, the complete transgene expression cassette from pCMX2.5-hIgG1Fc-XP was inserted into the pCSE vector backbone resulting in the vector pCSE2.5-hIgG1Fc-XP (Figure 1E).

### Test of the pCSE2.5-hIgG1Fc-XP vector platform for expressing various scFv-Fc antibodies

**Figure 8:**
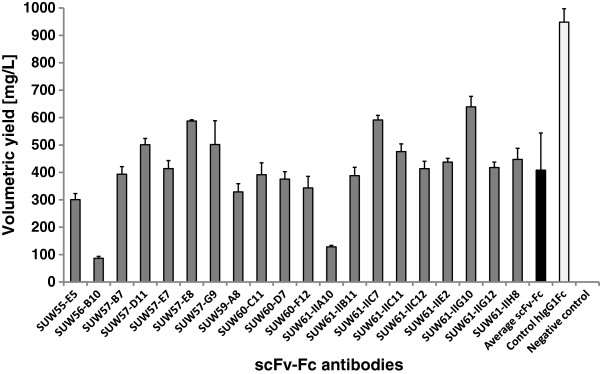
**Expression of 20 different scFv-Fc antibodies from the HAL phage display antibody generation pipeline.** A total of 20 different scFv antibody gene fragment isolated by phage display from the naïve antibody gene libraries HAL7/8 were subcloned into the vector pCSE2.5-hIgG1Fc-XP. The scFv-Fc antibodies were expressed after transient transfection in HEK293-6E cells in 25 mL scale and shake flasks. After 48 h one volume of fresh medium and 0.5% tryptone TN1 was supplemented. After 5 to 6 days supernatants were harvested and analyzed by human IgG/Fc capture ELISA. Standard deviations were calculated from a minimum of triplicate ELISA samples.

**Figure 9:**
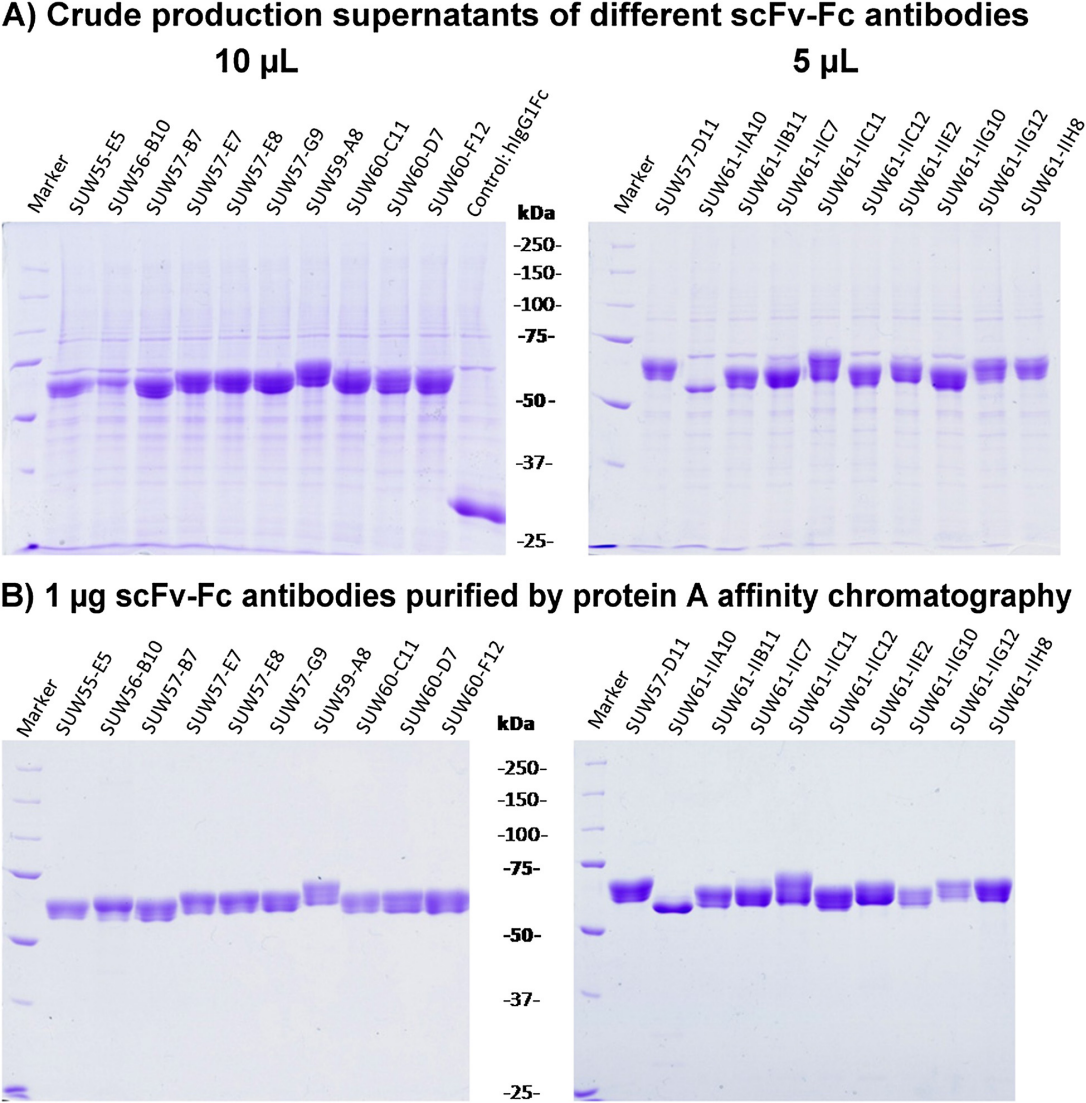
**Production supernatants and protein A purified samples of 20 different scFv-Fc antibodies. A**) Crude supernatant (10 μL or 5 μL) from 5 to 6 days production sample were analyzed by SDS-PAGE under reducing conditions and coomassie (R250) staining. **B**) Samples of 1 μg scFv-Fc antibody were also analyzed after protein A affinity purification.

More than 20 scFv gene fragments obtained by phage display from the HAL7/8 libraries were subcloned in frame into the Fc expression gene cassette of the vector pCSE2.5-hIgG1Fc-XP in order to produce IgG-like scFv-hIgG1Fc antibodies. Transient production using the standard transfection and production conditions resulted in volumetric yields of 100 to 600 mg/L scFv-Fc antibodies with an average of about 400 mg/L (Figure 8). The production levels of hIgG1-Fc without scFv even reached up to 900 mg/L. SDS-PAGE analyses of production supernatants revealed major protein bands corresponding to scFv-Fc antibodies with a molar mass of 55 to 60 kDa (Fc without scFv at 27 kDa) under reducing conditions. There was only a small amount of additional proteins released by the HEK293-6E (Figure 9A). Protein A affinity purification and desalting were sufficient to obtain all scFv-Fc antibodies in very high purify of >98% (Figure 9B).

**Figure 10:**
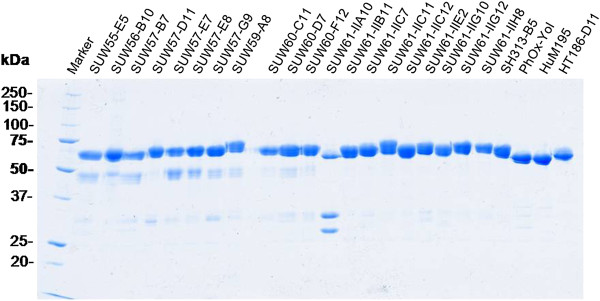
**Stability of the scFv-Fc antibodies produced in HEK293-6E.** A total of 1 μg of 24 different protein A purified scFv-Fc antibodies (scFv PhOx-Yol [15], HuM195 [16] and HT186-D11 [8] are not from the HAL antibody gene libraries) were analyzed after 6 months of storage in PBS without any protective protein or protease inhibitor. The concentration of the scFv-Fc was calculated immediately after protein A purification.

Storage of purified scFv-Fc antibodies for more than 6 months at 4°C in PBS without protective protein did not reveal any significant proteolytic degradation (Figure 10).

## Discussion

We previously described an efficient platform for the in vitro generation of recombinant human antibodies by phage display from the universal libraries HAL4/7/8 [4,51] which can address the required throughput even for proteome projects [1,3]. However, the production of milligram amounts of different antibodies for the end-user in an application compatible recombinant format created a new bottleneck in the overall-process. IgG-like bivalent scFv-Fc antibodies perform well in most immunoassays, but production of more complex antibody formats like scFv-Fc requires eukaryotic or even mammalian expression systems to achieve high yields of functional protein and to reduce the efforts during downstream processing [24].

In this study we optimized transient antibody expression in HEK293 cell lines in order to obtain high yields in combination with simple and robust protocols for production and downstream processing. We started with pCMV standard vectors and transient expression in HEK293T cells which already achieved more than 20 mg/L volumetric yields per day. However, laborious handling of these adherent cells, limited scalability, and daily medium exchange prevents this production system from being suitable for production of more than a few antibodies in parallel. In contrast, suspension cell lines allow easier handling, higher cell densities, and better scalability which would facilitate simultaneous production of dozens of antibodies as well as up-scaling to liter volumes in shake flasks or bioreactors.

The HEK293-6E cell line was developed to improve transient production of recombinant proteins [41,42,44,45,52] and used in this study. The first tests performed with the pCMV expression vector without oriP already resulted in volumetric yields of more than 140 mg/L scFv-Fc and 70 mg/L scFv-Fc-RNase after 5 days production which is about 10-fold higher than the expression levels in HEK293T. However, specific cellular production levels of HEK293T and HEK293-6E cells were in a similar range of 5–20 pg per cell per day. Therefore, the 5–10 fold higher production titers in HEK293-6E were mainly due to higher cell densities in suspension culture and the product accumulation over several days. In order to optimize antibody production in the HEK293-6E cell line, we generated test vectors without selection marker and with a short variant of EBV oriP, respectively. The elimination of the selection marker already resulted in a 2 to 2.5-higher production compared to the original pCMV vector in HEK293-6E which is probably due to the small size of the vector, *i.e.* the higher number of plasmid molecules per transfection, and the absence of any co-expressed selection marker protein. Co-expression of a fluorescent reporter protein resulted in an approximately 10-20% lower antibody yield (data not shown). The introduction of the short oriP variant into the expression vector pCMV-neo^-^ led to an additional increase of antibody production levels. The increased stability of plasmids containing the EBV oriP by episomal replication and more efficient distribution to the daughter cells during cell division in EBNA1^+^ cell lines is important for prolonged production but it cannot entirely explain the higher expression levels already after 72 hours which reached more than 250 mg/L for an CD30 specific scFv-Fc antibody and more than 100 mg/L for the corresponding scFv-Fc-RNase fusion protein. It was implicated that EBNA1 binding to oriP also increases the nuclear plasmid import and enhances the activity of strong constitutive promoters like the immediate early CMV promoter [53]. Moreover, binding of EBNA1 to the family of repeats (FR) domain of oriP activates the transcription of downstream EBV genes as well as transgenes [54,55].

The measurement of metabolic parameters revealed that glucose was almost entirely consumed after 3 days of production. In this case cells start to subsequently remetabolize L-lactate whereas lactate levels remained at a relatively low and constant level if glucose is fed in time. In contrast, L-glutamine was still not completely depleted. However, this is mainly due to the fact that our media were already supplemented with 7.5 mM of L-glutamine instead of the 4 mM of the original protocol. According to these results, we optimized and prolonged the fed batch shake flask cultivation by a doubling of culture volume by supplementation of medium, glucose and VPA in addition to our standard fed with casein hydrolysate TN1. Production titers improved almost twice for both, scFv-Fc and scFv-Fc-RNase constructs, after supplementation with one volume fresh medium in combination with TN1 48 h after transfection and a prolongation of the production to 7–8 days. Additional supplementation of glucose alone, performed 3 days after transfection, did result in just a small increase of the yield suggesting that the lack of glucose is not immediately causing detrimental effects on recombinant protein production as the metabolism of the cells is able to compensate this by using other components (*e.g.* L-lactate and L-glutamine). However, glucose supplementation maintained cell viability for at least one more day at a high level, thus minimizing the burden of contaminating, passively released intracellular proteins in the supernatant. In contrast, the combined addition of VPA and glucose 3 days after transfection increased the scFv-Fc-RNase production levels about 20 to 25%. VPA is known to induce total cellular protein expression like other histone deacetylase inhibitors, *e.g.* sodium butyrate [32]. Shake flask experiments supplemented with VPA/glucose had the highest glucose, L-lactate and L-glutamine concentrations remaining after 4–7 days of transient production. This appears to be comprehensible since further cell proliferation is substantially diminished after supplementation with VPA. However, addition of VPA seems to depend on other parameters because not all productions achieved higher antibody yields compared to the standard fed batches. Since VPA increases general protein expression [56,57], it may also alter cell properties, inducing apoptosis and potentially influence product quality. Therefore, the addition of VPA was not included into the standard protocol in the further study.

In order to combine all advantages of the previous vectors including compatibility to single step in frame subcloning of single chain antibody fragments from phage display libraries into the scFv-Fc antibody format, an entirely new transient mammalian expression vector was generated. The vector backbone was optimized for small size, compatibility to episomal replication in SV40 large T antigen or EBNA1 expressing mammalian cell lines, *i.e.* HEK293T and HEK293-6E, without any eukaryotic selection marker and high copy *E. coli* plasmid ori for efficient plasmid DNA preparation. Transient expression in HEK293-6E cells using these optimized pCSE vectors containing the scFv-Fc and scFv-Fc-RNase expression cassettes resulted in similar or better yields than the corresponding pCMV-oriP1 test vectors which achieved the highest production yields compared to all previous vectors. Moreover, reduced plasmid propagation in *E. coli* observed for the pCMV-neo^-^ and pCMV-oriP1 test vectors was overcome by employment of appropriate spacer sequences which separated the *E. coli* pMB1 ori from other DNA structure elements like the highly repetitive FR element of EBV oriP1 or the antibody gene expression cassette. Finally, an Fc expression cassette containing a NcoI/NotI in frame cloning cassette was introduced which allows single step cloning of scFv antibody gene fragments from phage display libraries like HAL4/7/8 into the scFv-Fc format. More than 20 different scFv antibody gene fragments were subcloned into the vector pCSE2.5-hIgG1Fc-XP and achieved average volumetric yields of 400 mg/L by transient production in HEK293-6E. Some scFv-Fc antibodies even exceeded production levels of 600 mg/L which is more than 4-5-fold higher than transient production levels of camelid heavy chain and single domain antibodies Fc proteins of 20 to 136 mg/L using the vector pTT5 in HEK293-6E cells [45]. Crude production supernatants contained more than 80% recombinant scFv-Fc antibody which facilitated protein A affinity purification. Almost all scFv-Fc antibodies were stable for more than half a year storage at 4°C without any protective agent. These scFv-Fc antibodies have been used successfully for many different immunoassays including ELISA, immunoblot and flow cytometry.

Improved production media and fed batch supplementation as well as controlled bioreactor processes may further increase cell densities and prolong production time which can further enhance production levels. Backliwal and colleagues [32] combined optimized PEI-based transfection at high cell densitities with the co-expression of cell cycle regulators p18 and p21, acidic fibroblast growth factor, VPA supplementation, consequent maintenance at high cell densities of 4x10^6^ cells/mL and upscaling to 2 L and achieved production levels of more than 1 g IgG in two weeks after transfection. However, individual optimization is not suitable for high-throughput production of non-characterized antibodies which requires a simple, robust and efficient protocol as we have shown in this study.

## Conclusion

Taken together, transient expression in HEK293-6E combined with optimized expression vectors and fed batch processes provides robust and versatile production of up to 600 mg/L recombinant IgG-like scFv-Fc antibodies. This expression system has been proven to be scalable from milliliter to liter volumes already in shake flasks which can address the production bottleneck of high throughput in vitro antibody generation pipelines. Moreover, efficient secretory production of more complex IgG-like scFv-Fc antibodies facilitates downstream processing and increases the compatibility to standard immunoassays and other applications compared to small recombinant antibody fragments. Concluding, efficient transient production of scFv-Fc antibodies and derivative fusion proteins in HEK293-6E provides the bridge from high throughput in vitro antibody generation to application which reduces efforts to obtain recombinant antibodies as highly specific detection reagents to all human proteins and protein variants in a proteome scale as well as to accelerate processes in therapeutic antibody development pipelines.

## Methods

### Cell culture

The adherently growing human embryonic kidney (HEK) cell line HEK293T/17 (HEK293T) was obtained from American type culture collection (ATCC, LGC Standards GmbH, Wesel, Germany: ATCC-No. CRL-11268) and cultured in Dulbecco’s modified Eagle’s medium (DMEM, high glucose (4.5 g/L) with 2 mM L-glutamine) supplemented with 8% (v/v) heat inactivated fetal calf serum (FCS) and 1% (v/v) of a 10.000 U/mL penicillin and 10 mg/mL streptomycin stock solution (all products from PAA, Pasching, Austria) at 37°C, 5% CO_2_ and 95% humidity.

The HEK293-6E cell line was licensed from National Research Council (NRC), Biotechnological Research Institute (BRI), Montreal, Canada [41]. Cells were cultured according to the supplier’s description in chemically defined F17 medium (Invitrogen, Life Technologies, Darmstadt, Germany) supplemented with 1 g/L pluronic F68 (Applichem, Darmstadt, Germany), 4 mM L-glutamine (PAA) and 25 mg/L G418 (PAA). HEK293-6E cells were cultivated in 125 mL to 1 L polycarbonate shake flasks (Corning, Amsterdam, The Netherlands) in a Minitron™ CO_2_ orbital shaker with 25 mm orbital (Infors, Bottmingen, Switzerland) at 37°C, 5% CO_2_ atmosphere and 110 rounds per minute (rpm) without exceeding 2×10^6^ cells/mL during maintenance. Small cultures were performed in 24 to 6 well multiwell plates using a linear shaker (Incutec K15-500) placed in a CO_2_ incubator with humidified atmosphere at 150 rpm.

### Construction of pCMV and pCMX plasmid vectors

The vector pCMV/myc/ER (Invitrogen) was used to construct the CD30 specific scFv-hIgG1Fc antibody and immunoRNase scFv-Fc-RNase as described previously [11]. The pCMV-HC (kindly provided by Dr. Thomas Jostock, now at Novartis) was originally generated by replacing the myc-tag and ER retention signal in pCMV/myc/ER by the Fc gene fragment encoding hinge, CH2 and CH3 domains of human IgG1 using NotI and XbaI cloning sites. The plasmid pCMV-HC was further modified to move the NcoI restriction site downstream to the end of the leader encoding the secretory signal peptide. Therefore, the KOZAK sequence [46] had to be modified from GCCACCATGG to GCCAACATGG without leaving the minimal consensus sequence ANNATGG. At the same time we further included the cloning sites PstI, PspOMI, BglII and NotI embedded into a short spacer sequence encoding nine amino acids of IGHV4 [58] and the five amino acid linker RSAAA. The leader sequence and new multiple cloning site was amplified from pCMV-HC with the desoxyoligonucleotides TS_pCMVFc_Leader_f1 and TS_pCMVFc_NheI_r1 (Table 2) and than subcloned into the PmlI and NheI of the same vector. The resulting plasmid pCMV-hIgG1Fc-XP was used for cloning the scFv gene fragments PhOxYol [15] and IIB6 [8] into the NcoI/NotI cloning site (pCMV-scFv-hIgG1Fc-PhOxYol and -IIB6). The corresponding immunoRNases were generated by cloning the gene fragment encoding a partial sequence the hIgG1-Fc fused with the RNase 1 from pCMV-scFv-hIgG1Fc-RNase-4E3 into these two vectors (pCMV-scFv-hIgG1Fc-RNase-PhOxYol and -IIB6). The vector pCMV-hIgG1Fc-XP was further optimized at the 5′ sequence of the Fc gene by replacing its SacII-XbaI fragment with PCR amplificates using the desoxyoligonucleotide forward primer TS_hIgG1CH2_SacII_f and the reverse primers TS_pCMV2.1_r, TS_pCMV2.2_r, TS_pCMV2.3_r, TS_pCMV2.4_r, TS_pCMV2.5_r, TS_pCMV3.2_r, and TS_pCMV4.2_r (Table 2). The resulting variants had different stop codons (and downstream nucleotides sequences), deletion of the 5′-terminal lysine codon (ΔK330), or the introduction of a BamHI fusion site replacing the carboxyterminal lysine by a serine codon (K330S) and were referred to as pCMVx.y-hIgG1Fc-XP (with x and y according to Table 2).

**Table 2:** Oligonucleotide primers used in this study

**Primer**	**5’→3’**	**REN**
TS_pCMVFc_Leader_f1	CTGGCACGTGGAAATTAATTAAACGCCGCCAACATGGGATGGAGCTGT	PmlI
TS_pCMVFc_NheI_r1	AGATGCTAGCGGCCGCAGATCTGGGCCCGGATTCCTGCAGCTGGACCTGTGCCATGGAGTGCGCGCCTGTGGAGAGA	NheI, NcoI
TS_hIgG1CH2_SacII_f	ACGGCGTGGAGGTGCATAATGCCAAGAC	
TS_pCMV2.1_r	AGCTTCTAGACTCATTTACCCGGGGACAGGGAGA	XbaI
TS_pCMV2.2_r	AGCTTCTAGACGCACTCATTTACCCGGGGACAGGGAGA	XbaI
TS_pCMV2.3_r	AGCTTCTAGAGCCGGCCGTCGCACTCATTTACCCGGGGACAGGGAGA	XbaI
TS_pCMV2.4_r	AGCTTCTAGATTCATTTACCCGGGGACAGGGAGA	XbaI
TS_pCMV2.5_r	AGCTTCTAGATTTATTTACCCGGGGACAGGGAGA	XbaI
TS_pCMV3.2_r	AGCTTCTAGACGCACTCATCCCGGGGACAGGGAGA	XbaI
TS_pCMV4.2_r	AGCTTCTAGACGCACTCAGGATCCCGGGGACAGGGAGA	XbaI
MHdCpCMV-NotI_f	GATCTGCGGCCGCTAGCGACAAAACTCACACATGCC	NotI
MHCH2-SacII_r	CTCCTCCCGCGGCTTTGTCTTGG	SacII
TS_oriP_PmeI-PvuII_f	GACTCAGCTGTTTAAA*C*GAACTAAACCTGACTACG	PvuII, PmeI
TS_oriP_FR_BsmBI/PciI_r	CACTCGTCTCACATGTATTCTCATGTTTGACAGC	BsmBI/PciI
TS_pUCori_MfeI_f	CAGTCAATTGGGATATCGCAGGAAAGAGCATGTGAGC	MfeI, EcoRV
TS_5’CMV_MfeI_r	CTGTCAATTGTGAATTCGATACCGATCTC	MfeI, EcoRI
TS_oriPspacer_Pci_r	CAACACATGTCAACATTAGCCCACCGTGC	PciI
TS_oriPspacer_Pml_f:	AGATCACGTGTAGGTGGGCGGGCCAAGA	PmlI

^a^ underlined: ribosomal binding site (modified KOZAK sequence).

Abbreviations: *REN* restriction endonuclease cloning site.

The pCMV vector family was further modified to eliminate the codons for the genetic upper hinge region (encoding the amino acids PKSC) which functionally belong to the CH1 including the cysteine involved in forming the interchain disulfide bond with the light chain in the Fab fragments. The modified Fc gene fragment was amplified from vector pCMV2.5-hIgG1Fc-XP with the primers MHdCpCMV-NotI_f and MHCH2-SacII_r and cloned into the NotI and SacII sites the same vector replacing the original Fc. The resulting vector was designated as pCMX2.5-hIgG1Fc-XP, respectively.

### Construction of test expression vectors compatible to expression in HEK293-6E cells

In order to optimize plasmids for the transient production in HEK293-6E cells two new test vector variants were generated expressing the antibody scFv-Fc-4E3 or its immunoRNase variant scFv-Fc-RNase-4E3 [11; 4E3 is referred to the CD30 specific scFv], respectively. The first vector variants were obtained by deleting the complete neomycin phosphotransferase (npt) selection marker cassette (including SV40 promoter/ori and Poly A signal) from the vectors pCMV-scFv-Fc-4E3 or pCMV-scFv-RNase-4E3 [11; originally termed pCMV-αCD30scFv-Fc and pCMV-αCD30scFv-RNase] using PvuII and blunted PciI resulting in the plasmids pCMVneo^-^-scFv-hIgG1Fc-4E3 and pCMVneo^-^-scFv-hIgG1Fc-RNase-4E3.

The second type of vectors were generated by introducing the short oriP amplified from the vector pTT5SH8Q2 (NRC, BRI) using the desoxyoligonucleotide primers TS_oriP_PmeI-PvuII_f and TS_oriP_FR_BsmBI/PciI_r (Table 2) using the PciI and PvuII cloning sites. The resulting vectors were referred to as pCMVoriP1-scFv-hIgG1Fc-4E3 and pCMVoriP1-scFv-hIgG1Fc-RNase-4E3.

### Construction of pCSE expression vectors compatible with our scFv antibody gene libraries

A minimal vector backbone consisting of beta lactamase gene (resistance to ampicillin) and the *E. coli* pUC ori was amplified from pCMX2.5-hIgG1Fc-XP using the desoxyoligonucleotide primers TS_pUCori_MfeI_f and TS_5′CMV_MfeI_r (Table 2). The PciI site was flanking the pUC ori was deleted. The PCR fragment was digested with MfeI and dephosphorylated. The complete EBV oriP was obtained from pCEP4 (Invitrogen) by restriction with MfeI und cloned into the amplified minimal vector backbone. The resulting vectors were referred to as pMfeI-oriP(+) or pMfeI-oriP(−) depending of the oriP1 orientation. The spacer fragment between dyad symmetry (DS) and family of repeats (FR) element of oriP was modified in order to remove the restriction sites Bsu36I, EcoRV, SpeI, MluI, NcoI, PspOMI and SpeI to minimize its size. The spacer fragment was amplified from pMfeI oriP(+) using the desoxyoligonucleotides TS_oriPspacer_Pci_r and TS_oriPspacer_Pml_f (Table 2) and digested with PciI (NcoI compatible overhang) und PmlI (blunt end). After cloning into the Bsu36I (blunted with Quick blunt Kit from NEB) and NcoI sites of pMfeI-ori(+) Bsu36I, PmlI, PciI and NcoI were removed and the resulting vector was referred to as pMfeI-oriP2(+). Moreover, a DNA sequence comprising bovine growth hormone (BGH) poly A signal (GenBank:AF117350.1: 75..282 bp), a 72 bp minimal SV40 ori/enhancer sequence (identical to human herpes viruses 1 and 5; GenBank:FJ593289.1: 134104..134196) comprising three critical elements 17-bp AT tract, central 23-bp perfect inverted repeat with four GAGGC boxes (P4/P3 and P2/P1), and 10-bp early palindrome EP [50,59] and appropriate spacer sequences as well as various restriction cloning sites was generated by gene sysnthesis (Mr. Gene, Regensburg, Germany) and cloned into the EcoRI and HpaI cloning sites of pMfeI-oriP2(+) resulting in the new vector pSV40oriP2(+). Finally, the CMV enhancer/promoter and complete gene expression cassettes of pCMX2.5-hIgG1Fc-XP, pCMV-scFv-Fc-4E3 or pCMV-scFv-RNase-4E3, respectively, were cloned into pSV40oriP2(+) using EcoRI and XbaI resulting in the vectors pCSE2.5-hIgG1Fc-XP, pCSE-scFv-hIgG1Fc-4E3 and pCSE-scFv-hIgG1Fc-RNase-4E3, respectively.

### Single step cloning of scFv gene fragments from HAL7/8 into pCSE2.5-hIgG1Fc-XP

In order to test the single step in frame cloning of scFv antibody gene fragments, more than 20 scFv gene fragments obtained by phage display to different antigens from the antibody gene libraries HAL 7/8 [4] were cloned into the NcoI/NotI cloning site of pCSE2.5-hIgG1Fc-XP to obtain scFv-hIgG1Fc antibody constructs. High quality plasmid preparations for transfection were done using the NucleoBond Xtra Midi Kit according to the manufacturer’s description (Machery Nagel, Düren, Germany).

### Transient transfection and production in HEK293T cells

Transient production in adherent HEK293T was done as previously described [38]. Briefly, HEK293T cells were seeded into 10 cm plates using 12.5 mL culture medium (Sarstedt, Nürnbrecht, Germany) to reach 70-80% confluency after day culture. To improve cell adherence during transfection and production, plates were prepared with sterile poly-L-lysine (Sigma-Aldrich, Munich, Germany; 0.01% (w/v), 75–150 kDa, cell-culture grade) for 15 minutes followed by to three washing steps with sterile phosphate buffered saline (PBS; 137 mM NaCl, 2.6 mM KCl, 8 mM Na_2_HPO_4_, 1.5 mM KH_2_PO_4_). Transfection was performed with 10 μg polyethyleneimide (PEI, Polysciences, Warrington, PA). A total of 10 μg high quality plasmid-DNA (Machery Nagel, Düren, Germany) and 80 μg/mL PEI were separately diluted in 600 μL DMEM. DNA and PEI solution were mixed and incubated for 15–30 min at room temperature (RT). DNA/PEI complexes were distributed over the cells and incubated for 24 h. Next day, medium was completely replaced by DMEM supplemented with 4% (v/v) IgG stripped FCS (PAA) and penicillin/streptomycin to minimize co-purification of bovine IgG by protein A/G affinity chromatography. The medium was usually harvested and exchanged everyday for up to two weeks. Production medium was harvested and exchanged every day for up to 1–2 weeks.

### Transient transfection and production in suspension HEK293-6E cells

Transient production in suspension HEK293-6E cells was performed as previously described [38]. Briefly, two days before transfection 5× 10^5^ cells/mL HEK293-6E were seeded in 25 to 150 mL serum free culture medium (without G418) into 125 to 1000 mL polycarbonate Erlenmeyer flasks with ventilation membrane caps (Corning, Amsterdam, The Netherlands) and incubated at 37°C and 110 rpm in a orbital shaker. For small scale productions 1 mL/well in a 12 well plate was incubated at 150 rpm in a linear shaker. A total of 1 μg high quality plasmid-DNA and 2.5 μg PEI per mL culture volume was prepared in 1/10 volume of fresh serum free culture medium as described above if not otherwise indicated. To quantify the transfection efficiency 1/20 of total DNA of the reporter plasmids pEF-FS-EGFP or pCS2-venus encoding the enhanced green fluorescence protein (EGFP) or the yellow fluorescence protein (YFP) variant venus [60], respectively, were added. After adding the DNA/PEI complexes, the cells were further cultured for 48 h. Then a final concentration of 0.5% (w/v) of tryptone N1 (TN1, Organotechnie S.A.S., La Courneuve, France) was added as described elsewere (Pham et al. 2005) together with one additional volume fresh culture medium.

### Protein A purification

All scFv-hIgG1Fc antibodies and scFv-hIgG1Fc-RNase constructs were purified by protein A affinity purification using 1 mL Bio-Scale Mini UNOsphere SUPrA Cartridges and the semiautomated Profinia 2.0 system (Biorad, Munich, Germany) according the standard manufacturer’s protocol.

### Flow cytometry

Transfection efficiency was measured either using a Guava EasyCyte mini flow cytometer (Guava Technologies, Hayward, CA, USA) or a FC500 flow cytometer (Beckman Coulter, Krefeld, Germany) usually 48 h after transfection. Transfected cells were detected by the co-expression of either GFP (pTTo/GFPq, NRC, BRI; 5% of total plasmid DNA) or YFP in the fluorescence light (FL) 1. Dead cells were stained with 2 μg/mL propidium iodide (PI, Sigma).

### Sodium dodecyl sulfate (SDS) polyacrylamide gel electrophoresis (PAGE)

SDS-PAGE was performed with a Mini-PROTEAN system (BioRad). Protein standards and samples were prepared under reducing conditions with Laemmli sample buffer for 5 minutes at 95°C or under non reducing conditions (Laemmli sample buffer without reducing agent) for 10 min at 65°C. Gels with up to 35 lanes were performed with a PerfectBlue Twin ExW S system (PEQlab, Erlangen, Germany). Coomassie staining was performed as described [14].

### Quantification of scFv-Fc antibodies by IgG/Fc capture ELISA and gel densitometry

The antibodies in the production supernatants were quantified by IgG/Fc capture ELISA as described [61]. Briefly, a total of 100 μL/well goat-anti-human immunoglobulin (polyvalent) capture antibody (Sigma, Cat.-No. I1761; 200 ng/mL) was diluted in PBS and coated in 96-well Maxisorp™ microtiter plates (Nunc, Thermo Fisher Scientific) for 1 h at 37°C. After blocking with 20% (v/v) FCS for 1 h at 37°C, the wells were washed three times with PBST (PBS with 0.05% [v/v] tween-20, Sigma) using a Columbus ELISA washer (TECAN, Crailsheim, Germany). A total of 100 μL/well of several sample dilutions and a half dilution series of the human IgG protein standard (N protein SL, Dade Behring) were prepared in PBS containing 1% (v/v) FCS and incubated for 1 h at 37°C. After washing, a total of 100 μL/well of 20 ng/mL goat-anti-human-IgG (Fc-specific) antibody horseradish peroxidase (HRP) conjugate (Sigma, Cat.-No. A0170) was incubated for 1 h at 37°C. After washing the color reaction was initiated with 100 μL/well 3,3′,5,5′-tetramethylbenzidine (TMB) substrate solution (20 volumes TMB solution A consisting of 30 mM potassium citrate, 0.5 mM citric acid, pH4.1 mixed with 0.5 volumes of TMB solution B consisting of 10 mM TMB in 10% [v/v] Aceton, 89% [v/v] ethanol, 0.3% [v/v] H_2_O_2_) and stopped by addition of 100 μL/well of 0.5 M H_2_SO_4_. The absorbance was measured at 450 nm against a reference wavelength of 620 nm (A_450_-A_620_) using the Sunrise ELISA reader (TECAN). Additionally, recombinant proteins produced in HEK293-6E cells under serum free culture conditions were quantified by SDS-PAGE after coomassie staining. Samples were prepared under reducing conditions and compared to purified standard proteins using the software image J (http://rsb.info.nih.gov/ij/disclaimer.html).

### Quantitative measurements of metabolic cell culture parameters

Glucose and L-lactate were quantified with a membrane-based enzymatic analyzer (YSI Model 2700 Select, Yellow Spring Instruments, Yellow Spring OH). The same instrument was also used to measure L-glutamine in combination with L-glutamic acid.

## Competing interests

The authors declare that they have no competing interests.

## Authors’ contributions

VJ and KB participated in the design of this study, supervised the production in HEK293-6E and the analysis of metabolic parameters, and drafted this part of the manuscript. AW performed productions in HEK293-6E cells and analyzed the metabolic parameters. SW carried out the comparative production of the more than 20 different scFv-Fc antibody clones in HEK293-6E cells and analyzed the data. MH provided the monoclonal recombinant human scFv antibodies and revised the manuscript. AF participated in the design of the new expression vectors and revised the manuscript. TS designed, coordinated and supervised this study, constructed most of the vectors, performed some productions, drafted and revised the manuscript. All authors read and approved the final manuscript.
